# A nomogram diagnostic prediction model of pancreatic metastases of small cell lung carcinoma based on clinical characteristics, radiological features and biomarkers

**DOI:** 10.3389/fonc.2022.1106525

**Published:** 2023-01-16

**Authors:** Jian-Xia Xu, Jin-Bao Hu, Xiao-Yan Yang, Na Feng, Xiao-Shan Huang, Xiao-Zhong Zheng, Qin-Pan Rao, Yu-Guo Wei, Ri-Sheng Yu

**Affiliations:** ^1^ Department of Radiology, The Second Affiliated Hospital of Zhejiang Chinese Medical University, Hangzhou, Zhejiang, China; ^2^ Department of Radiology, Second Affiliated Hospital, School of Medicine, Zhejiang University, Hangzhou, Zhejiang, China; ^3^ Precision Health Institution, General Electric (GE) Healthcare, Hangzhou, China

**Keywords:** pancreatic metastases, small cell lung carcinoma, pancreatic ductal adenocarcinomas, contrast-enhanced CT, nomogram

## Abstract

**Objective:**

To investigate clinical characteristics, radiological features and biomarkers of pancreatic metastases of small cell lung carcinoma (PM-SCLC), and establish a convenient nomogram diagnostic predictive model to differentiate PM-SCLC from pancreatic ductal adenocarcinomas (PDAC) preoperatively.

**Methods:**

A total of 299 patients with meeting the criteria (PM-SCLC n=93; PDAC n=206) from January 2016 to March 2022 were retrospectively analyzed, including 249 patients from hospital 1 (training/internal validation cohort) and 50 patients from hospital 2 (external validation cohort). We searched for meaningful clinical characteristics, radiological features and biomarkers and determined the predictors through multivariable logistic regression analysis. Three models: clinical model, CT imaging model, and combined model, were developed for the diagnosis and prediction of PM-SCLC. Nomogram was constructed based on independent predictors. The receiver operating curve was undertaken to estimate the discrimination.

**Results:**

Six independent predictors for PM-SCLC diagnosis in multivariate logistic regression analysis, including clinical symptoms, CA199, tumor size, parenchymal atrophy, vascular involvement and enhancement type. The nomogram diagnostic predictive model based on these six independent predictors showed the best performance, achieved the AUCs of the training cohort (n = 174), internal validation cohort (n = 75) and external validation cohort (n = 50) were 0.950 (95%CI, 0.917-0.976), 0.928 (95%CI, 0.873-0.971) and 0.976 (95%CI, 0.944-1.00) respectively. The model achieved 94.50% sensitivity, 83.20% specificity, 86.80% accuracy in the training cohort and 100.00% sensitivity, 80.40% specificity, 86.70% accuracy in the internal validation cohort and 100.00% sensitivity, 88.90% specificity, 87.50% accuracy in the external validation cohort.

**Conclusion:**

We proposed a noninvasive and convenient nomogram diagnostic predictive model based on clinical characteristics, radiological features and biomarkers to preoperatively differentiate PM-SCLC from PDAC.

## Introduction

Pancreatic metastases (PM) are rare tumor, accounting for 2-5% of all malignant tumors of the pancreas (1–3). PM are found more frequently at autopsy, with approximately 3-12% being found in patients with advanced cancer (4, 5). Common origins of PM neoplasms include the lung, kidney, breast, skin (especially melanoma), stomach and large intestine (6, 7). Although it is well known that there is less literature on lung cancer metastasis to the pancreas (6–8), small cell carcinoma more frequently develops pancreatic metastasis than adenocarcinoma or squamous cell carcinoma (9). It should be noted that pancreatic metastases of small cell lung carcinoma (PM-SCLC) frequently forms a single nodular lesion simulating a primary neoplasm of the pancreas (10). When there is no metastasis to other organs and the lesion is relatively small, it is often mistaken as pancreatic ductal adenocarcinoma (PDCA), its misdiagnosis rate is as high as 30% (10, 11).

Given the rarity of pancreatic metastases, it is difficult to accumulate sufficient data to justify any particular treatment and therefore there are no consensus guidelines for the treatment of pancreatic metastases (12). It has been reported that patients with PM-SCLC are often in the advanced stage of disease, with poor general condition or systemic metastasis, and low postoperative survival rate, and surgical treatment is not recommended (13). However, surgical resection is the only potential curative treatment for patients with PDAC and may be the main treatment modality to prolong patient survival and improve prognosis (14). Therefore, accurate preoperative diagnosis of PM-SCLC and differential diagnosis with PDAC are very important for clinical decision making and patient prognosis.

As a preoperative diagnostic method for pancreatic neoplasm, endoscopic ultrasound-guided fine needle aspiration (EUS-FNA) can clearly show the location and size of the tumor and confirm the pathological diagnosis. But preoperative biopsy in patients with potentially resectable tumor is controversial, because biopsy may cause the tumor to rupture or bleed, and may increase the risk of spread (15); Thus, it is clinically important and necessary to explore a noninvasive, reliable, and practical diagnostic prediction model of PM-SCLC.

Computed tomography (CT) is the most widely available and best­validated tool for imaging patients with pancreatic neoplasm, due to its advantages of non-invasiveness and convenience. CT provides good spatial resolution between tumor and background pancreatic parenchyma with wide anatomic coverage, and thus allowing comprehensive examination of local and distant disease in one single section (16). Multi­planar reconstruction on CT is important in tumor staging, providing selective visualization of important arterial and venous structures. This allows for precise visualization of the relationship of the primary tumor to the superior mesenteric artery (SMA), superior mesenteric vein (SMV) and coeliac axis thereby providing an assessment of vascular invasion and respectability (17). Although most experts acknowledge the added utility of MRI over CT in certain situations, including the main benefit in differentiating iso­attenuating pancreatic lesions and in characterization of indeterminate pancreatic lesions identified at prior CT examinations (15, 18). However, MRI is not widely used as the primary imaging modality in most centers due to issues of its cost and availability (18, 19).

With the development of imaging, significant progress has been made in the detection, diagnosis and evaluation of pancreatic malignancies, but there are limitations in the early diagnosis of PM-SCLC and PDAC, and the accuracy of single CT-enhanced examination for the differential diagnosis of PM-SCLC and PDAC is relatively low. In addition, serum level of tumor biomarkers, such as carcinoembryonic antigen (CEA), carbohydrate antigen 199 (CA199), and carbohydrate antigen 125 (CA125), are clinically reported markers that correlate with the development of malignant tumors. Domestic and international studies have shown that CEA is commonly used in the diagnosis of lung cancer. Higher preoperative CEA levels are associated with advanced or metastatic disease and therefore a poorer prognosis. Postoperative CEA does not return to normal or is elevated again suggesting residual or recurrent lesions (12, 20–22). In contrast, serum CA199, CA125 plays an important role in the development of PDAC and may be used as a biomarker for the diagnosis and/or prognosis of PDCA (23– 24). Therefore, it is clinically important to improve the differential diagnosis of PM-SCLC and PDAC with the help of simple and low-cost biomarkers of combined detection.

So far, there have been studies on differential diagnosis of PM and PDCA indexed in PubMed, but the results of these studies are not accurate enough. The characteristics of PM with different pathological types of malignancy are different, and no study of the differential diagnosis of single PM-SCLC and PDCA has been seen, and our sample size is relatively large. Previous PM studies focused on imaging feature analysis and did not include the study of clinical symptoms and biomarkers, and did not establish a prediction model with multicenter independent external validation.

This study aimed to develop a noninvasive, practical and intuitive nomogram model for preoperative diagnostic prediction of PM-SCLC using CT features combined with clinical symptoms and biomarkers for differential diagnosis with PDCA, and to evaluate its diagnostic efficiency by independent external validation.

## Materials and methods

### Patients

The study population was obtained from two independent hospitals. Between January 2016 and March 2022, a total of 8000 patients with pathologically confirmed small cell lung carcinoma with complete follow-up data were retrieved from the radiologic image archives of two participating institutions. Among them, 93 patients were diagnosed as pancreatic metastasis of lung cancer by pathological biopsy or CT examination. At the same time, 206 patients with pathologically confirmed PDAC were included. Overall, 299 patients with PM-SCLC or PDAC were enrolled in this retrospective study.

**Figure 1:**
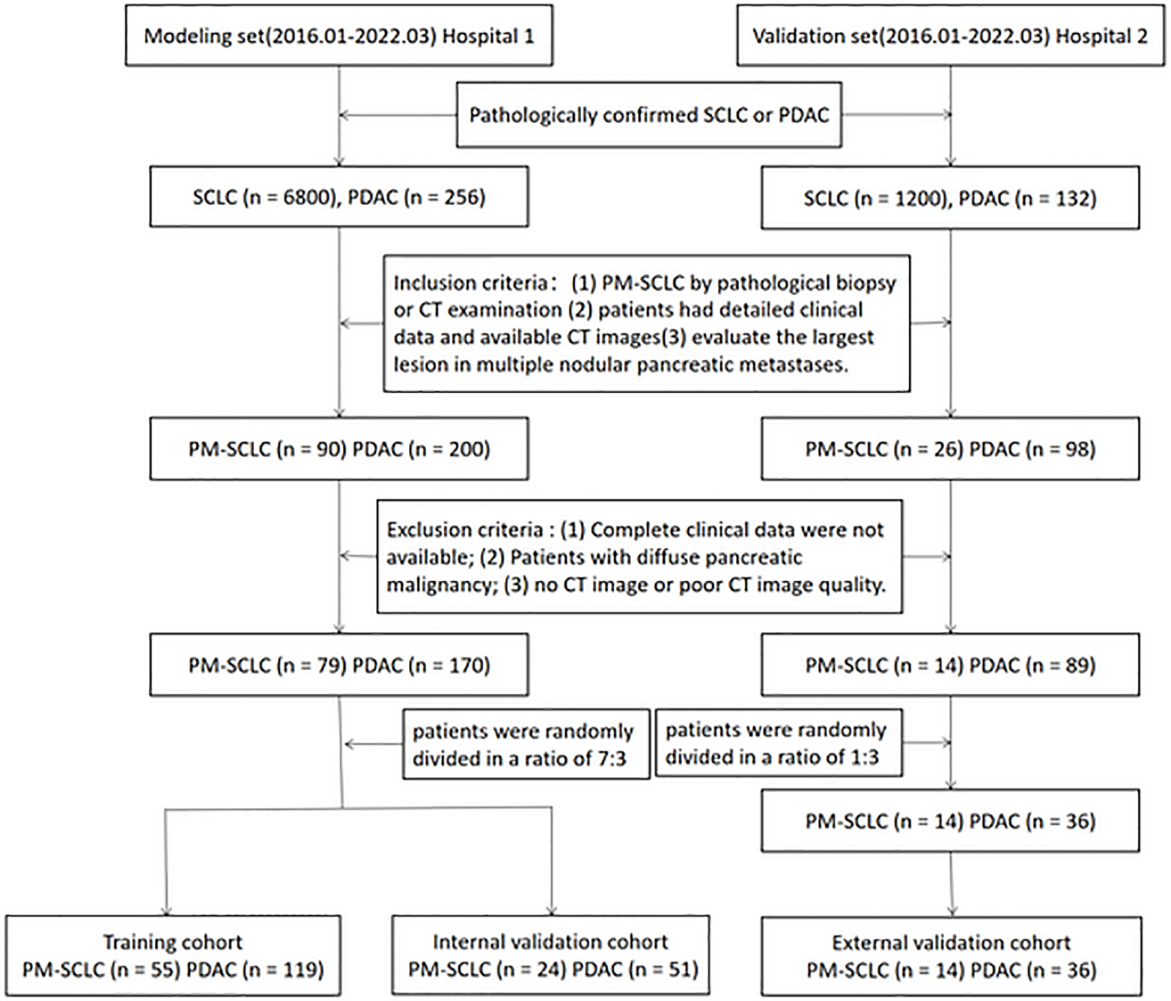
The flowchart of the patient selection.

A total of 249 patients with PM-SCLC (n = 79) or PDAC (n = 170) from the Second Affiliate Hospital of Zhejiang University Medical School (hospital 1), were randomly divided in a ratio of 7:3 into a training cohort (n = 174) and an internal validation cohort (n = 75), to determine the features representing independent risk for establishing the nomogram model and to verify the performance of the nomogram model. The independent external validation cohort consisted of 50 patients with PM-SCLC (n = 14) or PDAC (n = 36) from the Second Affiliated Hospital of Zhejiang Chinese Medical University (hospital 2), to verify the performance of the nomogram diagnostic predictive model. The inclusion criteria were as follows (1): patients with pathologically confirmed small cell lung cancer, in which pancreatic metastasis of lung cancer was confirmed by pathological biopsy or CT examination, and patients with pathologically confirmed PDAC; (2) patients had detailed clinical data and underwent CT and the image quality was satisfactory for analysis; (3) CT images with satisfactory quality contained non-enhanced phase, pancreatic phase and the portal venous phase; (4) CT examination was performed for the first detection of PM-SCLC; (5) evaluate the largest lesion in multiple nodular pancreatic metastases. The exclusion criteria were as follows: (1) complete clinical data were not available; (2) Patients with diffuse pancreatic malignancy; (3) no CT image or poor CT image quality. This retrospective multicenter study was approved by ethics committee of each participating hospital and waived the requirement of informed consent for all patients. The workflow of the patient selection process is given as **Figure 1**.

### CT acquisition

As this study was a multicenter retrospective design, a variety of CT scanners were used. Contrast-enhanced CT examination in hospital 1 was performed using multidetector-row CT (SOMATOM Definition Flash; Siemens Healthcare, Erlangen, Germany). Contrast-enhanced CT examination in hospital 2 was performed on two CT scanners: a Lightspeed VCT (GE Healthcare, Chicago, IL) and an Optima 540 (GE Healthcare). All patients were required to abstain from eating solid food for 4-6 hours before the examination. Patients were imaged in a supine position, and the scan range was from the diaphragmatic dome to the lower margin of the third lumbar spine. The CT parameters were as follows: detector configuration 128 x 0.6mm, tube voltage 120kVp, tube current 200mA, slice thickness 3mm, slice interval 3mm, pitch 0.6mm. The contrast agents in the two hospitals were Ultravist (Bayer Schering Pharma, Berlin, Germany) and Iohexol (Beijing North Road Pharmaceutical Co. Ltd., Beijing, China). A total of 100ml of nonionic iodinated contrast agent was administered with a pump injector at 3 mL/ss into an antecubital vein. The pancreatic phase and the portal venous phase were performed at 35s and 55s after the injection of contrast agent, respectively. The axial, coronal and sagittal images were reformatted with a 1.5-mm section thickness and a 1.5-mm interval after scanning.

### Clinical data and biomarker collection

All patients were performed with required examination. The clinical data and biomarker included age, gender, clinical symptoms, CEA value, CA125 value, CA199 value, CEA (≥5ug/L), CA125 (≥35Ku/L), CA199 (≥37Ku/L).

### Image analysis

All original images were interpreted by two experienced abdominal radiologists (with 15 and over 20 years of experience, respectively) independently and retrospectively, who were blinded to pathological results and clinical information of each patient. In cases of initial disagreement, the two radiologists discussed findings to consensus.

The variables of CT imaging were as follows: tumor size (maximum diameter on axial images, the unit is mm), number (one, two, equal to or more than three lesions in the pancreas), location (head, neck, body or tail), tumor shape (round, lobulated or irregular), necrosis (presence or absence), margin (well-defined or ill-defined), parenchymal atrophy (presence or absence), retention cyst (presence or absence), peripancreatic fluid (presence or absence), vascular involvement [presence or absence), common bile duct dilatation (> 8 mm (25)], pancreatic duct through (presence or absence), pancreatic duct dilatation (≥ 3 mm (26)), pushed pancreatic duct (presence or absence), coeliac enlarged lymph nodes [short-axis diameter was larger than 10 mm or included necrosis of any size (27, 28)], peritumoral lymph nodes (none, 0<SD<8mm, SD≥8mm), relative density (low density, iso density, high density), blood supply (poor, rich), enhancement pattern (homogeneous, heterogeneous), enhancement degree (none, mild, moderate, strong), enhancement type (gradual, fast forward and backward, other), enhancement type1 (circular, overall, partial).

Tumor shape was defined as round (more than 80% of the transverse section had a circular or oval appearance without an angular shape), lobulated (more than 2 protrusions and the length of the protrusion was longer than 1/3 of the maximum diameter of the lesion) or irregular (more than 20% of the transverse section appeared to have a non quasi circular or angular shape). Necrosis was considered present when non-enhancing or hypo-attenuating foci with a CT attenuation value of 0-20 Hounsfield Units (HU) were observed within the tumor. Relative density was defined as lower, equal, or higher than that of normal pancreatic parenchyma in the non-enhanced phase. Blood supply was defined as lower or higher than that of normal pancreatic parenchyma in the Pancreatic phase. Enhancement patterns were defined as follows: homogeneous enhancement indicated that the difference between the most strongly and weakly enhanced portion of the lesions was less than 10 HU, or indicated heterogeneous enhancement. The degree of enhancement was quantitatively judged by the difference between the post-enhancement CT attenuation value and the non-enhanced CT attenuation value. If the difference was < 5 HU, the tumor was considered to exhibit a none enhancement pattern; the difference was < 20 HU, the tumor was considered to exhibit a mild enhancement pattern; 20-40 HU was considered to represent a moderate enhancement pattern, and > 40 HU was judged to be a strong enhancement pattern. Enhancement type 1 were evaluated in the portal venous phase. Difference value of 1 (the Pancreatic phase values minus the non-enhanced phase values), difference value 2 (the portal venous phase CT values minus the non-enhanced phase values), and difference value 3 (the portal venous phase CT values minus the pancreatic phase values) were calculated. CT attenuation values of the parenchyma in all lesions were measured in HU using a 20 mm^2^ circular region-of-interest (ROI). The ROI cursors were placed so as to encompass as much of the most strongly enhanced portion of the tumor as possible and to avoid necrosis, cystic degeneration, and vascular travel area in tumor and adjacent structures. The quantitative analysis was tested three times for each feature and the averaged values. were used.

### Model establishment and evaluation

Multi-class classification model was constructed using a transformed logistic regression. We used the extended logistic regression method penalized by LASSO with 10-fold cross-validation to train the best performing classification models from the training cohort prior to internal and external validation. To investigate the classification power of finally retained clinical, radiological features and biomarkers, three multi-class models were developed for the diagnosis and prediction of PM-SCLC: clinical model, CT imaging model, and combined model. For assessing the performance of diagnostic prediction models, the receiver operating characteristic (ROC) curves were displayed in the training, internal validation, and external validation cohort, respectively. The performance metrics such as sensitivity, specificity, accuracy and the area under the curve (AUC) were calculated.

### Statistical analysis

Before analyses, variables with zero variance were excluded from analyses. For missing data, mode imputation was used for categorical variables, and mean imputation was used for continuous variables. Finally, the data were standardized by the standardization. Data distributions were measured using the Kolmogorov-Smirnov test or the Shapiro-Wilk test. Numerical data was presented as the mean (standard deviation (SD)) and categorical data was shown as frequency (percentages). Student’s t test was used for continuous variables with normal distribution, while Mann-Whitney U test was applied for data with non-normal distribution and the chi-square or Fisher’s exact test was used for categorical variables. Variables presented statistically significant in logistic univariate analysis were obtained into a multivariate logistic regression analysis and a backward stepwise approach was used to identify independent predictors of PM-SCLC. Then, the best logistic model was built by using the extended logistic regression method penalized with LASSO with 10-fold cross-validation from the established optimal feature subsets of the training cohort, and a logistic-based nomogram was performed.

Calibration was assessed using the Hosmer-Lemeshow goodness-of-fit test, and P > 0.05 indicated insignificant deviance from the theoretical perfect calibration in the training and validation sets. The receiver operating characteristic (ROC) curves were used to evaluate the model performance, and the discriminatory ability of the model was evaluated through the area under the ROC curve (AUC), and sensitivity, specificity, accuracy and area under curve (AUC) were calculated. We performed decision curve analysis (DCA) to visualize the net benefit for clinical decisions.

All statistical analyses for the present study were performed by using IBM SPSS (version 26.0), R statistical software (version 3.5.1) and Python (version 3.5.6). A two-tailed p-value <0.05 indicated statistical significance.

## Results

### Baseline characteristics

The baseline patient characteristics were shown in **Table 1**. In our study, a total of 249 patients from the hospital 1 were randomly divided in a ratio of 7:3 into a training cohort (n = 174) and an internal validation cohort (n = 75), and the independent external validation cohort consisted of 50 patients from the hospital 2. There were no significant differences in the variables of clinical characteristics, CT features and biomarkers among the three cohorts.

**Table 1:** Baseline characteristics of datasets.

Characteristics	Training cohort(n=174)	Internal validation cohort(n=75)	External validation cohort(n=50)	P
Age (mean (SD))	61.9 (9.5)	63.2 (10.0)	64.6 (8.9)	0.189
Gender (%) Female Male	64 (36.8) 110 (63.2)	19 (25.3) 56 (74.7)	14 (28.0) 36(72.0)	0.569
Clinical symptoms (%) Absence Presence	86 (49.4) 88 (50.6)	41 (54.7) 34 (45.3)	19 (38.0) 31 (62.0)	0.128
CEA value (mean (SD))	47.2 (271.6)	19.1 (77.0)	26.1 (119.2)	0.770
CA125 value (mean (SD))	122.8 (245.9)	99.5 (246.4)	87.1 (121.4)	0.387
CA199 value (mean (SD))	1691.4 (3725.7)	1847.9 (3500.9)	2053.4 (3670.7)	0.215
CEA (%) **<5** **≥5**	105 (60.3) 69 (39.7)	41 (54.7) 34 (45.3)	32 (64.0) 18 (36.0)	0.587
CA125 (%) **<35** **≥35**	109 (62.6) 65 (37.4)	38 (50.7) 37 (49.3)	29 (58.0) 21 (42.0)	0.996
CA199 (%) **<37** **≥37**	58 (33.3) 116 (66.7)	27 (36.0) 48 (64.0)	13 (26.0) 37 (74.0)	0.386
Tumor size (mean (SD))	31.8(16.1)	33.0(17.1)	31.4(14.4)	0.803
Number (%) One Two Equal to or more than 3	159 (91.4) 10 (58.8) 5 (2.9)	70 (93.3) 1 (1.3) 4 (5.4)	49 (98.0) 0 (0.0) 1 (2.0)	0.260
Location (%) Head Neck Body Tail	57 (32.8) 27 (15.5) 41 (23.6) 49 (28.2)	23 (30.7) 17(22.7) 14 (18.6) 21 (28.0)	19 (38.0) 6(12.0) 14(28.0) 11(22.0)	0.486
Tumor shape (%) Round Lobulated Irregular	104 (59.8) 11 (63.2) 59 (33.9)	45 (60.0) 3 (4.0) 27 (36.0)	29 (58.0) 1 (2.0) 20 (40.0)	0.481
Necrosis (%) Absence Presence	107 (61.5) 67 (38.5)	42 (56.0) 33 (44.0)	30 (60.0) 20 (40.0)	0.719
Margin (%) well-defined Ill-defined	52 (29.9) 122 (70.1)	24 (32.0) 51 (68.0)	22 (44.0) 28 (56.0)	0.150
Parenchymal atrophy (%) Absence Presence	105 (60.3) 69 (39.7)	73 (97.3) 2 (2.7)	46 (92.0) 4 (8.0)	0.991
Retention cyst (%) Absence Presence	163 (93.7) 11 (6.3)	37 (49.3) 38 (50.7)	28 (56.0) 22 (44.0)	0.66
Peripancreatic fluid (%) Absence Presence	158 (90.8) 16 (9.2)	67 (89.3) 8 (10.7)	44 (88.0) 6 (12.0)	0.803
Vascular involvement (%) Absence Presence	92 (52.9) 82 (47.1)	37 (49.3) 38 (50.7)	28 (56.0) 22 (44.0)	0.699
Common bile duct dilatation (%) Absence Presence	140 (80.5) 34 (19.5)	64 (85.3) 11 (14.7)	38 (76.0) 12 (24.0)	0.437
Pancreatic duct through (%) Absence Presence	169 (97.1) 5(2.9)	70(93.3) 5 (6.7)	49 (98.0) 1 (2.0)	0.312
Pancreatic duct dilatation (%) Absence Presence	169 (97.1) 5(2.9)	70(93.3) 5 (6.7)	49 (98.0) 1 (2.0)	0.285
Pushed Pancreatic duct (%) Absence Presence	159 (91.4) 15(8.6)	66(88.0) 9 (12.0)	40 (80.0) 10 (20.0)	0.063
Coeliac enlarged lymph nodes (%) Absence Presence	126 (72.4) 48(27.6)	48(64.0) 27 (36.0)	39 (78.0) 11(22.0)	0.324
Peritumoral lymph nodes (%) none 0<SD<8mm SD≥8mm	102 (58.6) 57 (32.8) 15 (8.6)	41 (54.7) 21 (28.0) 13 (17.3)	30 (60.0) 17 (34.0) 3 (6.0)	0.536
Relative density (%) low density iso density high density	102 (58.6) 67 (38.5) 5 (2.9)	42 (56.0) 28 (37.3) 5 (6.7)	24 (48.0) 25 (50.0) 1 (2.0)	0.268
blood supply (%) poor rich	166 (95.4) 8 (4.6)	73 (97.3) 2 (2.7)	49 (98.0) 1 (2.0)	0.780
Enhancement pattern (%) Homogeneous Heterogeneous	61 (35.1) 113 (64.9)	27 (36.0) 48 (64.0)	18 (36.0) 32 (64.0)	0.999
Enhancement degree (%) none Mild Moderate Strong	4 (2.3) 49 (28.2) 75 (43.1) 46 (26.4)	2 (2.7) 27 (36.0) 30 (40.0) 16 (21.3)	1 (2.0) 14 (28.0) 23 (46.0) 12 (24.0)	0.965
Enhancement type (%) Gradual Fast forward and backward Other	129 (74.1) 44 (25.3) 1 (0.6)	55 (73.3) 16 (21.3) 4 (5.4)	43 (86.0) 5 (10.0) 2 (4.0)	0.148
Enhancement type 1 (%) Circular Overall partial	102 (58.6) 57 (32.8) 15 (8.6)	41 (54.7) 21 (28.0) 13 (17.3)	30 (60.0) 17 (34.0) 3 (6.0)	0.065
Nonenhanced phase (mean (SD))	36.4 (6.8)	35.0 (7.8)	36.1 (7.4)	0.892
Pancreatic phase (mean (SD))	65.6 (20.3)	60.8 (21.5)	63.0 (20.9)	0.734
Portal venous phase (mean (SD))	69.2(21.4)	64.0(20.5)	68.2(2.3)	0.75
Difference value 1(mean (SD))	29.2 (18.1)	25.8 (17.7)	26.9 (18.8)	0.485
Difference value 2(mean (SD))	32.8 (18.8)	29.0 (16.9)	32.1 (18.4)	0.886
Difference value 3(mean (SD))	3.6 (10.8)	3.2 (8.8)	5.2 (7.9)	0.207

CEA, carcinoembryonic antigen; CA199, carbohydrate antigen 199; CA125, carbohydrate antigen 125. Data are means (standard deviations).

### Comparison of patient characteristics between PM-SCLC and PDAC

A comparison of patient characteristics between PM-SCLC and PDAC groups in the three cohort was summarized in **Table 2**. The training cohort of 174 patients included 55 PM-SCLC (31.6%) and 119 PDAC (68.4%). The internal validation cohort of 75 patients included 24 PM-SCLC (32.0%) and 51 PDAC (68.0%). External validation cohort of 50 patients included 14 PM-SCLC (28.0%) and 36 PDAC (72.0%). There were significant differences in clinical symptoms, CA199, location, necrosis, margin, parenchymal atrophy, vascular involvement, common bile duct dilatation, pancreatic duct dilatation between the two groups, in the three cohorts, according to univariate analysis (P < 0.05). In addition, similar tendencies were observed for gender, CEA value, CA125 value, CA199 value, tumor size, number, tumor shape, retention cyst, peripancreatic fluid, peritumoral lymph nodes, enhancement pattern, enhancement degree, enhancement type, enhancement type1 between the two groups, respectively, in the three cohorts, although not always statistically significant in univariate analysis.

**Table 2:** Comparison of patient characteristics between PM-SCLC and PDAC groups in the three cohorts.

Characteristics	Training cohort (n=174)			Internal validation cohort(n=75)			External validation cohort(n=50)		
	PM-SCLC(n=55); PDAC(n=119)			PM-SCLC(n=24); PDAC(n=51)			PM-SCLC(n=14); PDAC(n=36)		
	PM-SCLC	PDAC	p	PM-SCLC	PDAC	p	PM-SCLC	PDAC	p
Age (mean (SD))	60.0 (8.6)	62.8 (9.8)	0.071	59.8 (9.4)	64.7 (10.0)	***0.047***	62.2 (9.3)	65.5 (8.7)	0.249
Gender (%) Female Male	12 (21.8) 43 (78.2)	52 (43.7) 67 (56.3)	***0.024***	6(25.0) 18 (75.0)	13 (25.5) 38 (74.5)	0.964	1 (7.1) 13(92.9)	13 (36.1) 23(63.9)	***0.041***
Clinical symptoms (%) Absence Presence	51 (92.7) 4 (7.3)	35 (29.4) 84 (70.6)	***<0.001***	24 (100.0) 0 (0.0)	17 (33.3) 34 (66.7)	***<0.001***	13 (92.9) 1 (7.1)	6 (16.7) 30 (83.3)	***<0.001***
CEA value (mean (SD))	42.6 (132.9)	8.2 (15.2)	***0.017***	127.6 (476.5)	9.4 (13.6)	0.079	71.9 (120.2)	8.3 (11.9)	0.307
CA125 value (mean (SD))	121.9(268.0)	89.1(236.3)	***0.010***	176.0(360.24)	97.7(166.7)	0.201	111.3(105.8)	77.7 (127.1)	0.385
CA199 value (mean (SD))	404.7 (1650.1)	2514.9 (3912.0)	***<0.001***	529.7(2215.5)	2238.1(4163.3)	0.064	94.6 (150.2)	2815.1 (4089.5)	<0.001
CEA (%) <5 ≥5	29 (52.7) 26 (47.3)	76 (63.9) 43 (36.1)	0.108	11 (45.8) 13 (54.2)	30 (58.8) 21 (41.2)	0.292	8 (57.1) 6 (42.9)	24 (66.7) 12 (33.3)	0.529
CA125 (%) <35 ≥35	34 (61.8) 21(38.2)	75 (63.0) 44 (37.0)	0.385	9 (37.5) 15 (62.5)	29 (56.9) 22 (43.1)	0.118	7 (50.0) 7 (50.0)	22 (61.1) 14 (38.9)	0.475
CA199 (%) <37 ≥37	33 (60.0) 22(40.0)	25 (21.0) 94 (79.0)	***<0.001***	13 (54.2) 11 (45.8)	14 (27.5) 37 (72.5)	***0.025***	9 (64.3) 5 (35.7)	4 (11.1) 32(88.9)	***<0.001***
Tumor size (mean (SD))	28.1(18.1)	33.5(13.1)	***<0.001***	21.3(13.0)	38.5(16.1)	***<0.001***	27.2(16.4)	33.0(13.4)	0.207
Number (%) One Two Equal to or more than 3	40 (72.7) 10 (18.2) 5 (9.1)	119 (100.0) 0 (0.0) 0 (0.0)	***<0.001***	20 (83.3) 0 (0.0) 4 (16.7)	50 (98.0) 1 (2.0) 0 (0.0)	***0.009***	13 (92.9) 1 (7.1) 0 (0.0)	36 (100.0) 0 (0.0) 0 (0.0)	0.105
Location (%) Head Neck Body Tail	11 (20.0) 4 (7.3) 13 (23.6) 27 (49.1)	46 (38.7) 23 (19.3) 28 (23.5) 22 (18.5)	***<0.001***	1 (4.2) 6(25.0) 8 (33.3) 9 (37.5)	22 (43.1) 11(21.6) 6 (11.8) 12 (23.5)	***0.004***	0 (0.0) 2(14.3) 5(35.7) 7(50.0)	19 (52.8) 4(11.1) 9(25.0) 4(11.1)	***0.002***
Tumor shape (%) Round Lobulated Irregular	41 (74.5) 2 (3.6) 12 (21.8)	63 (52.9) 9 (7.6) 47 (39.5)	***0.002***	19 (79.2) 1 (4.2) 4 (16.7)	26(51.0) 2 (3.9) 23(45.1)	0.054	11 (78.6) 0 (0.0) 3 (21.4)	18 (50.0) 1 (2.8) 17 (47.2)	0.175
Necrosis (%) Absence Presence	44 (80.0) 11 (20.0)	63 (52.9) 56 (47.1)	***<0.001***	20 (83.3) 4 (16.7)	21 (41.2) 30 (58.8)	*0.001*	12 (85.7) 2 (14.3)	18 (50.0) 18 (50.0)	***0.021***
Margin (%) well-defined Ill-defined	25 (45.5) 30 (54.5)	27 (22.7) 92 (77.3)	***<0.001***	14 (58.3) 10 (41.7)	10 (19.6) 41 (80.4)	***0.001***	14 (100.0) 0 (0.0)	8(22.2) 28 (77.8)	***<0.001***
Parenchymal atrophy (%) Absence Presence	50 (90.9) 5 (9.1)	55 (46.2) 64 (53.8)	***<0.001***	24 (100.0) 0 (0.0)	18 (35.3) 33 (64.7)	***<0.001***	14 (92.0) 0 (8.0)	16 (44.4) 20 (55.6)	***<0.001***
Retention cyst (%) Absence Presence	55 (100.0) 0 (0.0)	108 (90.8) 11 (9.2)	***0.027***	24 (100.0) 0 (0.0)	49 (96.1) 2 (3.9)	0.325	14 (56.0) 0 (44.0)	32 (88.9) 4 (11.1)	0.193
Peripancreatic fluid (%) Absence Presence	54 (98.2) 1 (1.8)	104 (87.4) 15 (12.6)	***0.018***	23 (95.8) 1 (4.2)	44 (86.3) 7 (13.7)	0.211	14 (100.0) 0 (0.0)	30 (83.3) 6 (16.7)	0.103
Vascular involvement (%) Absence Presence	47 (85.5) 8 (14.5)	45 (37.8) 74 (62.2)	***<0.001***	22 (91.7) 2 (8.3)	15 (29.4) 36 (70.6)	***<0.001***	14 (100.0) 0 (0.0)	14 (38.9) 22 (61.1)	***<0.001***
Common bile duct dilatation (%) Absence Presence	51 (92.7) 4 (7.3)	89(74.8) 30 (25.2)	***<0.001***	24 (100.0) 0 (0.0)	40 (78.4) 11 (21.6)	***0.014***	14 (100.0) 0 (0.0)	24 (66.7) 12 (33.3)	***0.013***
Pancreatic duct through (%) Absence Presence	51 (92.7) 4(7.3)	118 (99.2) 1(0.8)	0.107	22(91.7) 2 (8.3)	48(94.1) 3 (5.9)	0.691	14 (100.0) 0(0.0)	35 (97.2) 1 (2.8)	0.529
Pancreatic duct dilatation (%) Absence Presences	44 (80.0) 11(20.0)	54 (45.4) 65(54.6)	***<0.001***	23(95.8) 1 (4.2)	22(43.1) 29 (56.9)	***<0.001***	13 (92.9) 1 (7.1)	10 (28.6) 25 (71.4)	***<0.001***
Pushed Pancreatic duct (%) Absence Presence	41 (74.5) 14(25.5)	118 (99.2) 1(0.8)	***<0.001***	15(62.5) 9 (37.5)	51(100.0) 0 (0.0)	***<0.001***	4 (28.6) 10 (71.4)	36 (100.0) 0 (0.0)	***<0.001***
Coeliac enlarged lymph nodes (%) Absence Presence	37 (67.3) 18(32.7)	89 (74.8) 30(25.2)	0.613	16(66.7) 8 (33.3)	32(62.7) 19 (37.3)	0.741	7 (50.0) 7(50.0)	32 (88.9) 4(11.1)	0.203
Peritumoral lymph nodes (%) none 0<SD<8mm SD≥8mm	48 (87.3) 5 (9.1) 2 (3.6)	54 (45.4) 52 (43.7) 13 (10.9)	***<0.001***	22 (91.7) 2 (8.3) 0 (0.0)	19 (37.3) 19 (37.3) 13 (25.5)	***<0.001***	10 (60.0) 3 (34.0) 1 (6.0)	20 (60.0) 14 (34.0) 2 (6.0)	0.504
Relative density (%) low density iso density high density	33 (60.0) 22 (40.0) 0 (0.0)	69 (58.0) 45 (37.8) 5 (4.2)	0.664	12 (50.0) 10(41.7) 2 (8.3)	30 (58.8) 18 (35.3) 3 (5.9)	0.759	6 (42.9) 8 (57.1) 0 (0.0)	18 (50.0) 17 (47.2) 1 (2.8)	0.707
blood supply (%) poor rich	50 (90.9) 5 (9.1)	116 (97.5) 3 (2.5)	0.357	24 (100.0) 0 (0.0)	49 (96.1) 2 (3.9)	0.325	13 (92.9) 1 (7.1)	36 (100.0) 0 (0.0)	0.105
Enhancement pattern (%) Homogeneous Heterogeneous	21 (38.2) 34 (61.8)	40 (33.6) 79 (66.4)	0.112	13 (54.2) 11 (45.8)	14 (27.5) 37 (72.5)	***0.025***	9 (64.3) 5 (35.7)	9 (25.0) 27 (75.0)	***0.009***
Enhancement degree (%) Without Mild Moderate Strong	0 (0.0) 18 (32.7) 28 (50.9) 9 (16.4)	4 (3.4) 31 (26.1) 47 (39.5) 37 (31.1)	***0.004***	0 (0.0) 13 (54.2) 10 (41.7) 1 (4.2)	2 (3.9) 14 (27.5) 20 (39.2) 15 (29.4)	***0.028***	0(0.0) 4 (28.6) 9 (64.3) 1 (7.1)	1 (2.8) 10 (27.8) 14 (38.9) 11 (30.6)	0.095
Enhancement type (%) Gradual Fast forward and backward Other	29 (52.7) 26 (47.3) 0 (0.0)	100 (84.0) 18 (15.1) 1 (0.8)	***<0.001***	15 (62.5) 8 (33.3) 1 (4.2)	40 (78.4) 8 (15.7) 3 (5.9)	***0.005***	9 (64.3) 4 (28.6) 1 (7.1)	34 (94.4) 1 (2.8) 1 (2.8)	***0.007***
Enhancement type 1 (%) Circular Overall partial	13 (23.6) 28 (50.9) 14 (25.5)	17 (14.3) 76 (63.9) 26 (21.8)	***0.030***	8 (33.3) 13(54.2) 3 (12.5)	5 (9.8) 28 (54.9) 18 (35.3)	0.016	1 (7.1) 13 (92.9) 0(0.0)	5 (13.9) 25 (69.4) 6 (16.7)	0.179
Nonenhanced phase (mean (SD))	36.9 (7.7)	36.2 (6.3)	0.357	36.1 (9.5)	34.5 (6.5)	0.402	38.2 (6.9)	35.2 (7.5)	0.202
Pancreatic phase (mean (SD))	64.4 (15.5)	66.6(22.3)	0.818	57.9 (17.8)	62.2 (23.0)	0.419	61.6 (14.8)	63.5 (23.1)	0.771
Portal venous phase (mean (SD))	66.1(16.6)	70.7(23.2)	0.165	60.5(15.9)	65.7(22.2)	0.307	65.8(15.9)	69.1(23.3)	0.629
Difference value 1(mean (SD))	27.6 (12.6)	30.0 (13.6)	0.427	21.8 (12.0)	27.7 (17.6)	0.175	23.4 (11.9)	28.3 (19.8)	0.410
Difference value 2(mean (SD))	29.2 (14.2)	34.5 (18.3)	0.068	24.4 (11.4)	31.2 (18.6)	0.102	27.6 (12.6)	33.9 (20.0)	0.282
Difference value 3(mean (SD))	1.7 (11.3)	4.5 (10.5)	0.071	2.6 (9.9)	3.5 (8.3)	0.688	4.2 (7.9)	5.6 (8.0)	0.597

CEA, carcinoembryonic antigen; CA199, carbohydrate antigen 199; CA125, carbohydrate antigen 125. Data are means (standard deviations).

P values ≤ 0.05 written in bold and italics indicates a statistically significant difference between two groups.

### Feature selection and model construction

**Figure 2:**
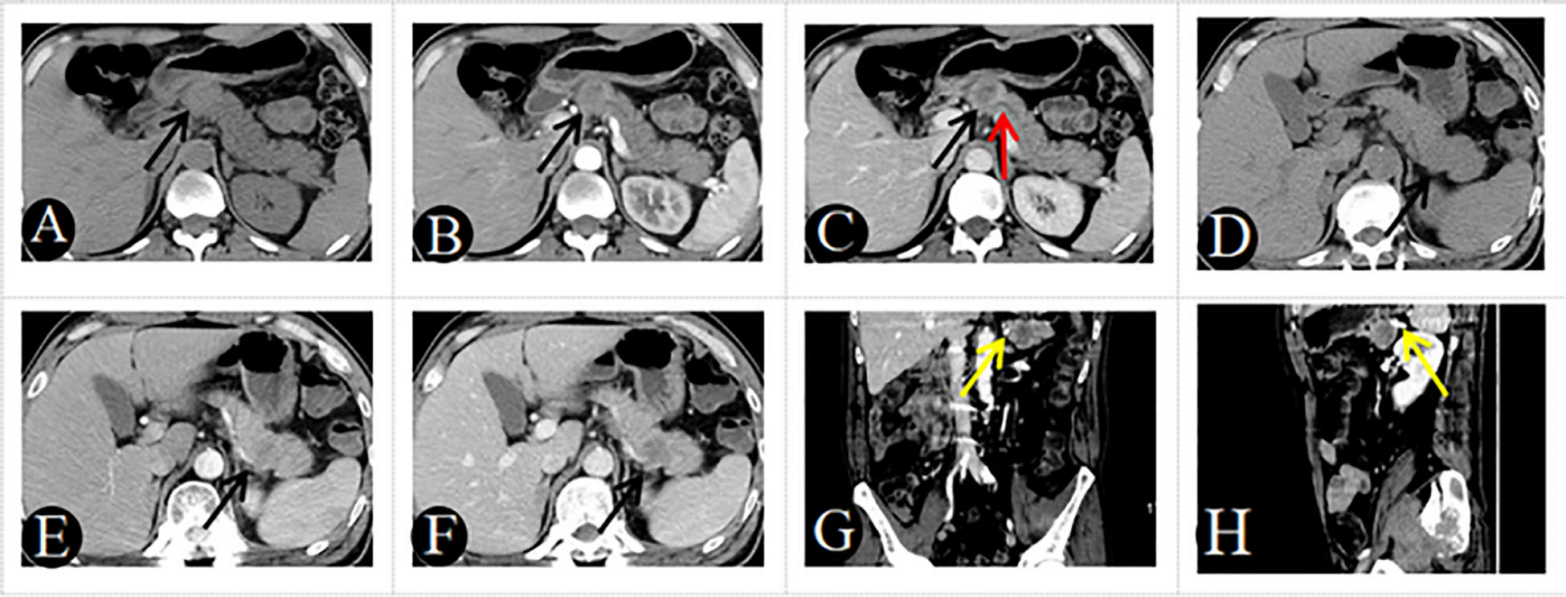
A 48-year-old man with PM-SCLC, absence of clinical symptoms, CA199 negative. Axial unenhanced **(A)**, pancreatic parenchymal **(B)**, and portal **(C)** phases CT images, demonstrated a low- or equal-attenuation nodules approximately 28mm in diameter in the neck of the pancreas (black arrow). The lesion showed gradual enhancement type, the typical rim enhancement and the pushed pancreatic duct (C, red arrow), with neither pancreatic parenchymal atrophy nor vascular invasion. A 51-year-old man with PM-SCLC, absence of clinical symptoms, CA199 negative. Axial unenhanced **(D)**, pancreatic parenchymal **(E)**, and portal **(F)** phases CT images, demonstrated a low- or equal-attenuation nodules approximately 30mm in diameter in the tail of the pancreas (black arrow). The lesion showed fast forward and backward enhancement type and the absence of vascular invasion (**G, H**, yellow arrow).

**Figure 3:**
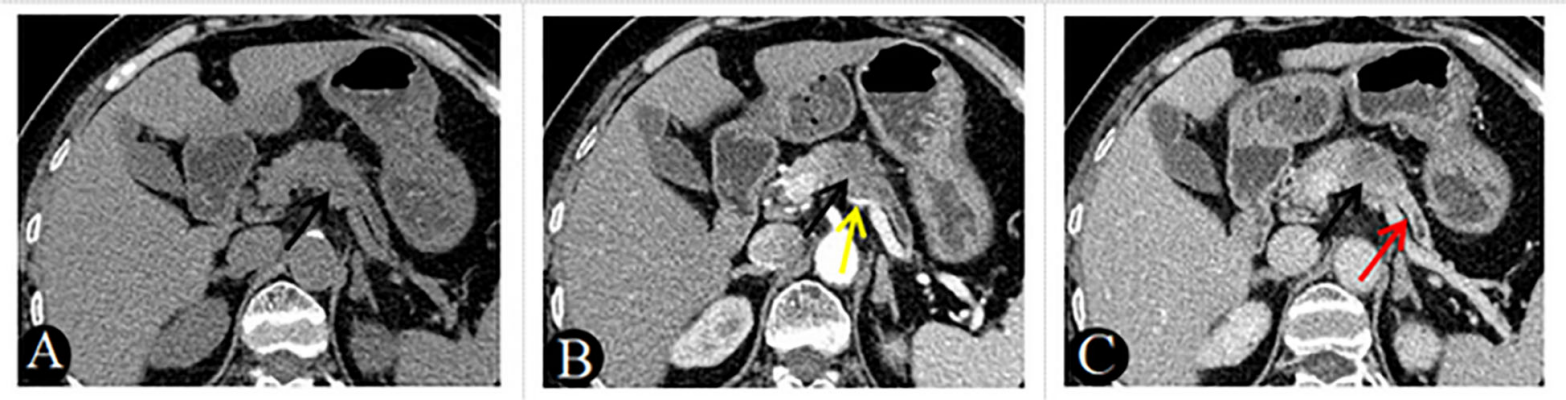
A 69-year-old woman with PDAC, absence of clinical symptoms, CA199 positive. Axial unenhanced **(A)**, pancreatic parenchymal **(B)**, and portal **(C)** phases CT images, demonstrated a low-attenuation nodules approximately 25mm in diameter in the neck of the pancreas (black arrow). The lesion showed gradual enhancement type and ill-defined margins, associated with main pancreatic duct dilatation (**C**, red arrow) and atrophy of the body and tail of the pancreas **(A–C)**. Note also the involvement of the splenic artery, which is narrowed (**B**, yellow arrow).

The algorithm of extended logistic regression penalized by LASSO finally determined three clinical characteristics and biomarkers (clinical symptoms, CA199, gender) in the **Table 3**, 6 radiological features (pancreatic duct dilatation, pushed pancreatic duct, tumor size, parenchymal atrophy, vascular involvement, enhancement type) in the **Table 4**, 6 independent predictors (clinical symptoms, CA199, tumor size, parenchymal atrophy, vascular involvement, enhancement type) in the **Table 5** for PM-SCLC diagnosis distinguishing from PDAC, respectively. Three models (clinical model, CT imaging model, and combined model) were constructed considering not only single-modal features but also the fusion of multi-modal features for the diagnosis and prediction of PM-SCLC (**Figures 2**, **3**).

**Table 3:** Three clinical characteristics and biomarkers for PM-SCLC diagnosis in multivariate logistic regression analysis.

variables	OR	95% CI		P
		Lower	Upper	
Clinical symptoms	0.031	0.010	0.100	***< 0.001***
CA199	0.191	0.076	0.476	***< 0.001***
Gender	3.165	1.243	8.059	***0.016***

P values ≤ 0.05 written in bold and italics indicates a statistically significant difference between two groups.

**Table 4:** Six radiological features for PM-SCLC diagnosis in multivariate logistic regression analysis.

variables	P	OR	95% CI	
			Lower	Upper
Pancreatic duct dilatation	***0.010***	41.219	2.427	699.953
Pushed Pancreatic duct	***0.004***	52.362	3.471	789.895
Tumor size	***0.002***	1.869	1.259	2.777
Parenchymal atrophy	***0.013***	0.221	0.067	0.731
Vascular involvement	***< 0.001***	0.067	0.019	0.233
Enhancement type	***< 0.001***	3.232	1.728	6.045

P values ≤ 0.05 written in bold and italics indicates a statistically significant difference between two groups.

**Table 5:** Six optimal independent predictors for PM-SCLC diagnosis in multivariate logistic regression analysis.

variables	P	OR	95% CI	
			Lower	Upper
Clinical symptoms	***< 0.001***	0.023	0.004	0.087
CA199	***0.003***	0.164	0.045	0.519
Tumor size	***0.032***	1.053	1.007	1.107
Pancreatic atrophy	***0.001***	0.094	0.019	0.360
Vascular involvement	***< 0.001***	0.065	0.011	0.286
Enhancement type	***0.005***	2.856	1.429	6.248

P values ≤ 0.05 written in bold and italics indicates a statistically significant difference between two groups.

### Performance of logistic models

The combined models demonstrated good diagnostic predictive performance for PM-SCLC in three logistic models, as demonstrated in **Table 6**. For training cohort, the sensitivities of clinical, CT imaging, and combined models were 56.4%, 76.4%, and 94.5%, respectively; the specificities of clinical, CT imaging, and combined models were 83.1%, 85.6%, and 83.2%, respectively; and the accuracies of clinical, CT imaging, and combined models were 82.2%, 85.2%, and 86.8%, respectively. For internal validation cohort, the sensitivities of clinical, CT imaging, and combined models were 54.2%, 79.2%, and 100.0%, respectively; the specificities of clinical, CT imaging, and combined models were 83.2%, 80.4%, and 80.4%, respectively; and the accuracies of clinical, CT imaging, and combined models were 77.3%, 80.0%, and 86.7%, respectively. For external validation cohort, the sensitivities of clinical, CT imaging, and combined models were 50.0%, 100.0%, and 100.0%, respectively; the specificities of clinical, CT imaging, and combined models were 86.2%, 89.2% and 88.9%, respectively; and the accuracies of clinical, CT imaging, and combined models were 84.4%,90.1% and 92.7%, respectively, (**Table 6**).

**Table 6:** Performance of three models for PM-SCLC.

	Training cohort			Internal validation cohort			External validation cohort		
	Sensitivity	Specificity	Accuracy	Sensitivity	Specificity	Accuracy	Sensitivity	Specificity	Accuracy
Clinical	56.4%	83.1%	82.2%	54.2%	83.2%	77.3%	50.0%	86.2%	84.4%
CT imaging	76.4%	85.6%	85.2%	79.2%	80.4%	80.0%	100.0%	89.2%	90.1%
Combined	94.5%	83.2%	86.8%	100.0%	80.4%	86.7%	100.0%	88.9%	92.7%

ROC values of three logistic models for PM-SCLC in three cohort, were shown in the **Table 7**. For training cohort, the AUCs of clinical, CT imaging, and combined models were 0.898 (95%CI, 0.857-0.937), 0.915 (95%CI, 0.869-0.955), and 0.950 (95%CI, 0.917-0.976), respectively; For internal validation cohort, the AUCs of clinical, CT imaging, and combined models were 0.875 (95%CI, 0.809-0.931), 0.917 (95%CI, 0.859-0.962), and 0.928 (95%CI, 0.873-0.971), respectively; For external validation cohort, the AUCs of clinical, CT imaging, and combined models were 0.944 (95%CI, 0.894-0.985), 0.996 (95%CI, 0.985-1.000), and 0.976 (95%CI, 0.944-1.000), respectively.

**Table 7:** ROC values of three models for PM-SCLC.

	Training cohort(n=174)		Internal validation cohort(n=75)		External validation cohort(n=50)	
	AUC	95%CI	AUC	95%CI	AUC	95%CI
Clinical model	0.898	(0.857-0.937)	0.875	(0.809-0.931)	0.944	(0.894-0.985)
CT imaging model	0.915	(0.869-0.955)	0.917	(0.859-0.962)	0.996	(0.985-1.000)
Combined model	0.950	(0.917-0.976)	0.928	(0.873-0.971)	0.976	(0.944-1.000)

**Figure 4:**
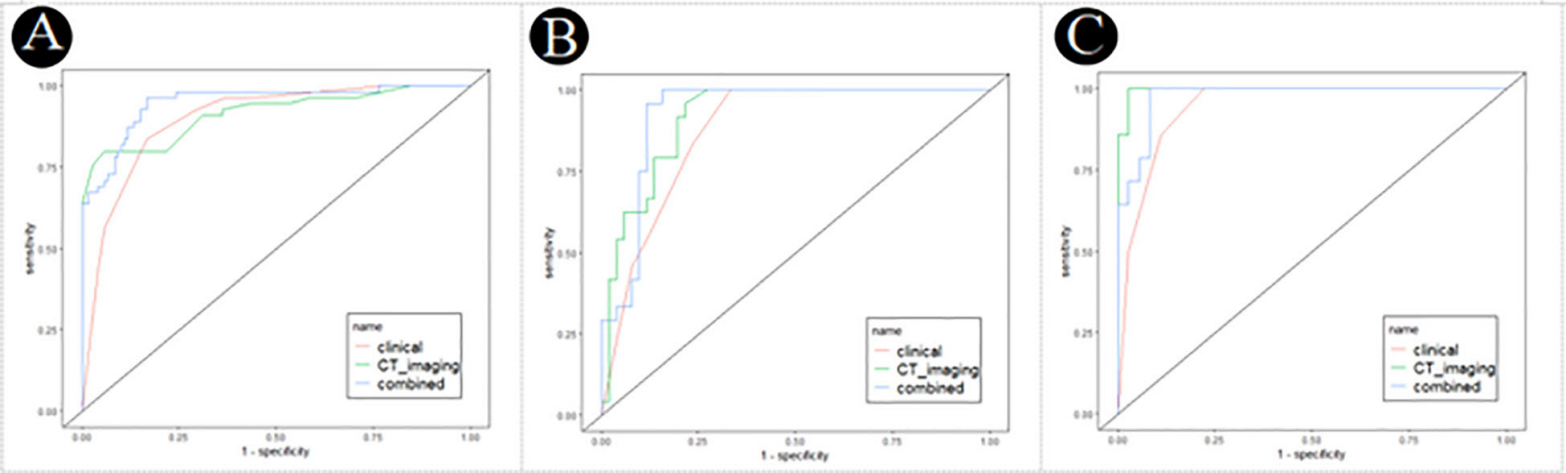
Receiver operating characteristic (ROC) curves of the clinical, CT imaging and combined model for PM-SCLC in three cohorts. **(A)** The training cohort. **(B)** The internal validation cohort. **(C)** The external validation cohort.

By validating and comparing the diagnostic predictive power of the three logistic models, the combined model displayed the highest in the training cohort and internal validation cohort, while the combined model had slightly higher diagnostic predictive power than the clinical model and slightly lower than the CT imaging model in the external validation cohort (**Table 7**, **Figure 4**).

**Figure 5:**
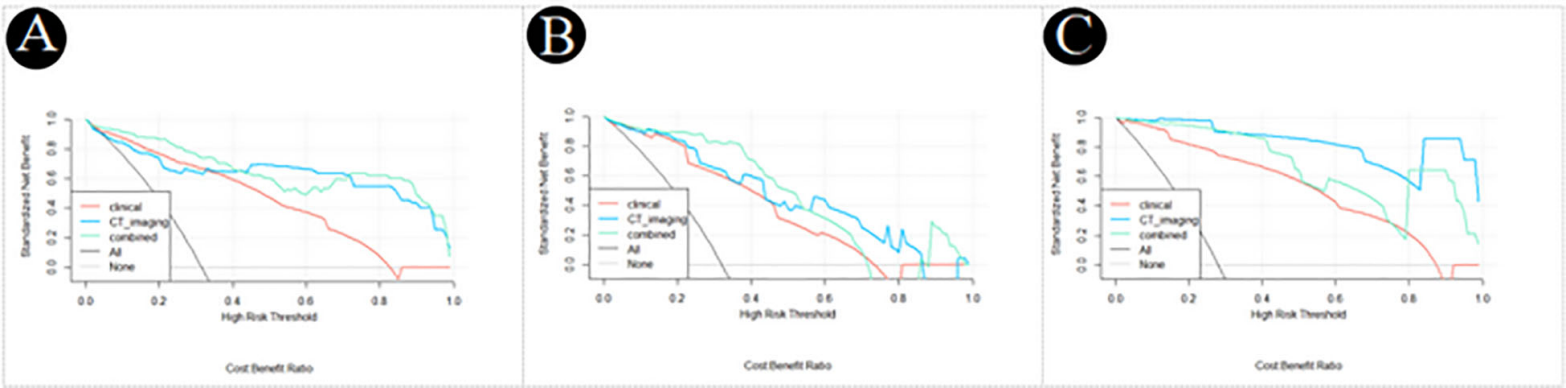
Decision curve analysis of the clinical, CT imaging and combined model for PM-SCLC in three datasets. **(A)** The training cohort. **(B)** The internal validation cohort. **(C)** The external validation cohort.

The decision curve analysis of the clinical, CT imaging and combined model for PM-SCLC in three cohorts (**Figure 5**), indicated that the three logistic models all had had high net benefit. The net benefit for clinical decisions of the combined model displayed the highest in the training cohort and internal validation cohort, while the combined model had slightly less than the CT imaging model in the external validation cohort. Therefore, the combined model was the most clinically useful, valuable, and safest of diagnostic prediction model for PM-SCLC, although not always had the greatest net benefit in three cohorts.

### Nomogram establishment

**Figure 6:**
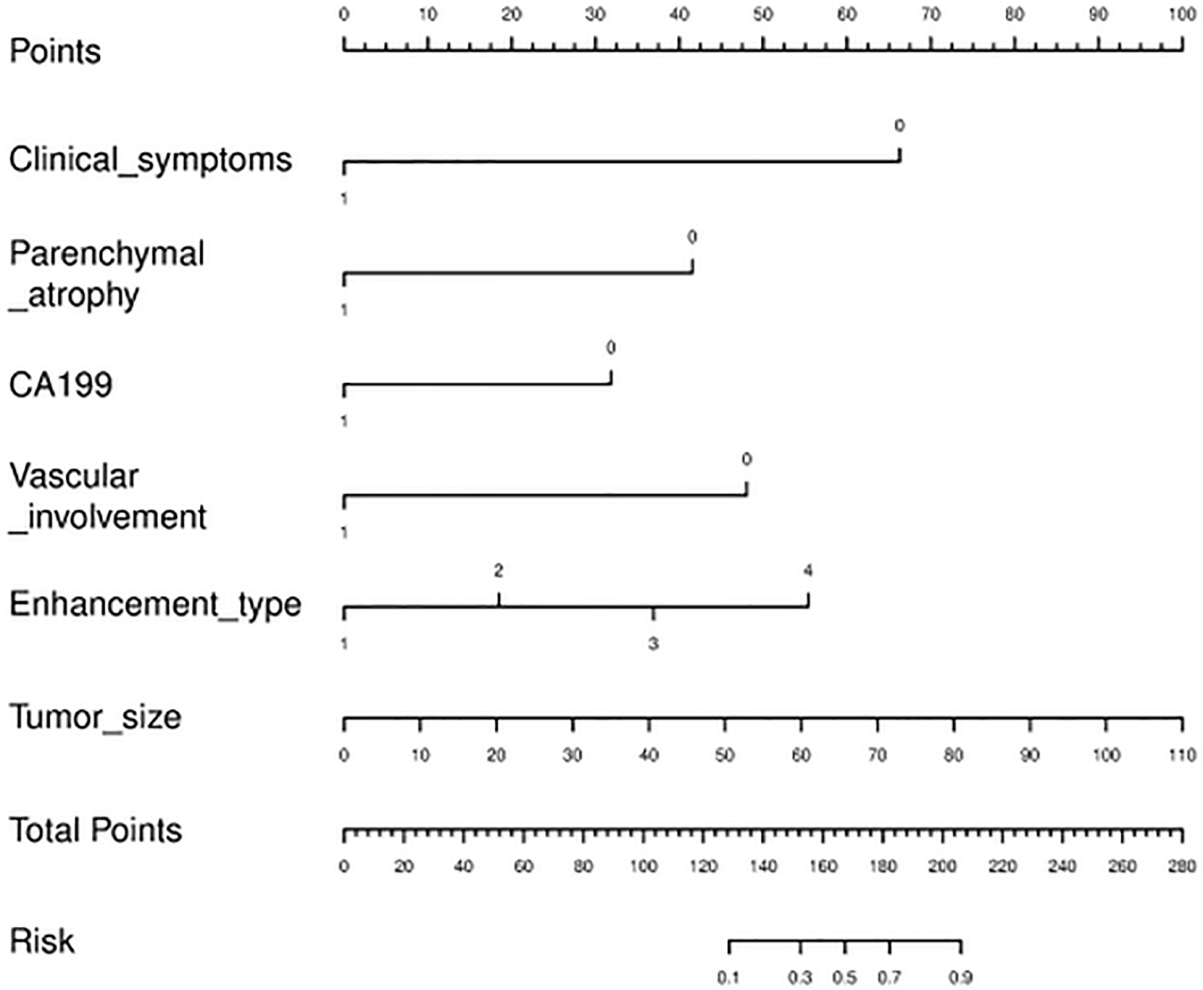
The nomogram diagnostic predictive model of PM-SCLC.

**Figure 7:**
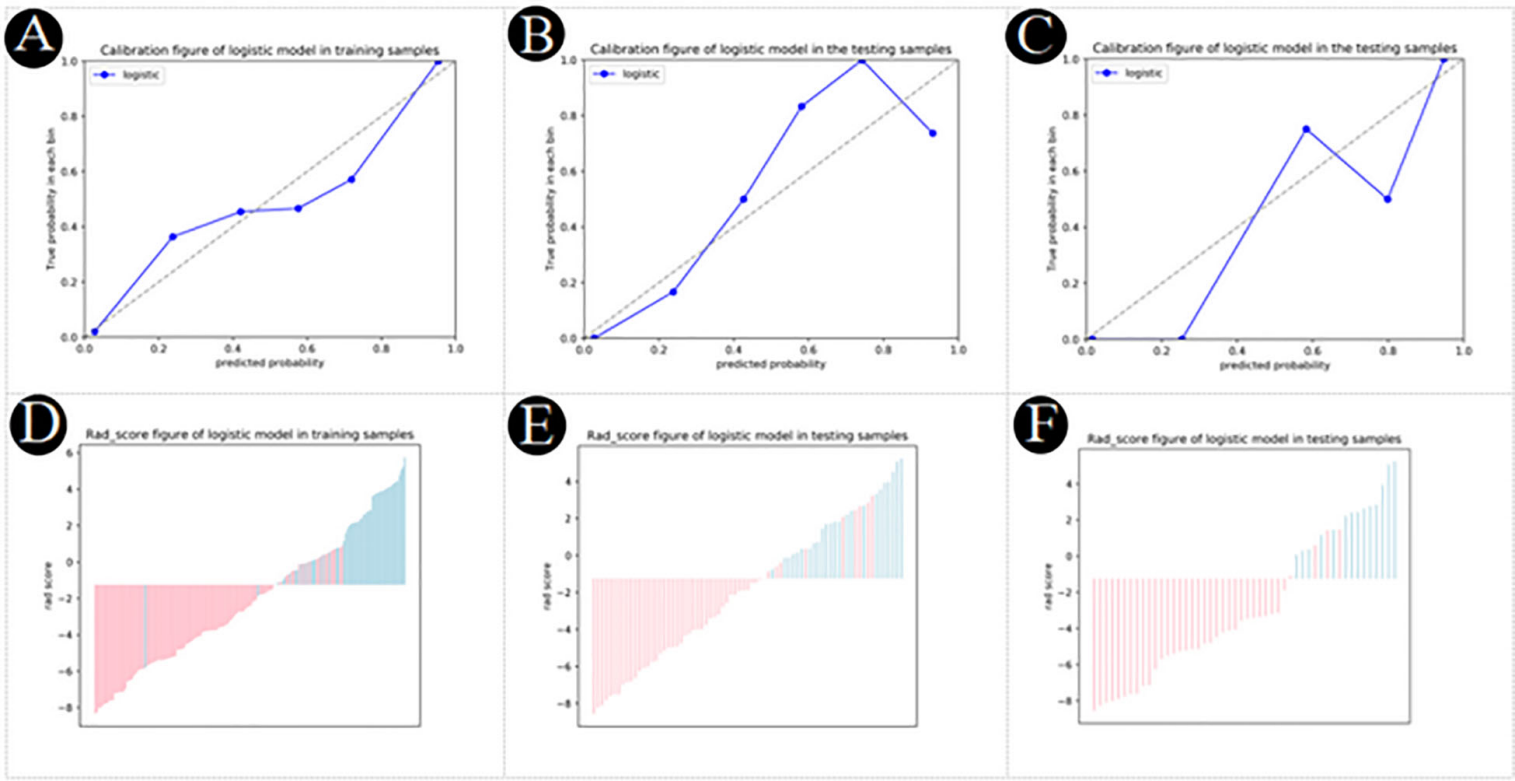
**(A–C)** Calibration plot of logistic model for PM-SCLC in the training cohort, the internal validation cohort, the external validation cohort, respectively. **(D–F)** Rad score plot of logistic model for PM-SCLC in the training cohort, the internal validation cohort, the external validation cohort, respectively.

The nomogram diagnostic predictive model of PM-SCLC was created based on the best logistic model, which was built by using the extended logistic regression method penalized with LASSO with 10-fold cross-validation from the established optimal feature subsets of the training cohort (**Figure 6**). The calibration curves of the combined nomogram showed good calibration performances in the training cohort, internal validation cohort, and external validation cohort, the high agreements between ideal curves and calibration curves were observed (**Figures 7A–C**). The rad score plots of the combined nomogram showed good discrimination between PM-SCLC and PDAC in the training cohort, internal validation cohort, and external validation cohort (**Figures 7D–F**).

## Discussion

In this study, a convenient nomogram diagnostic predictive model was established and validated to differentiate PM-SCLC from PDAC preoperatively. The nomogram model included six optimal independent predictors (clinical symptoms, CA199, tumor size, pancreatic atrophy, vascular involvement and enhancement type) for PM-SCLC diagnosis in multivariate logistic regression analysis, which were easily obtained and quantified. It was relatively reliable and easy-to-use for the clinicians and radiologists and was established based on clinical characteristics, radiological features and biomarkers.

Pancreatic metastasis of lung cancer is rare, accounting for less than 2% of all pancreatic malignancies (24, 29), the majority being small-cell lung carcinoma (SCLC) (25). In previous studies on the differential diagnosis of PM from PDAC (18, 26), in addition to small cell lung cancer, the primary tumors of these metastases also included non-small cell lung cancer, renal carcinoma, breast cancer, melanoma, gastrointestinal cancer, sarcoma (28). However, the characteristics of these PM depended on the primary tumor and were shown to be single, multiple, or diffuse, hypovascular or hypervascular on CT or MRI. And the results of these studies have some limitations, such as the inclusion of blood-poor and blood-rich metastases in the same cohort compared with PDAC, which may induce selection bias. Therefore, we differentiated PM-SCLC and PDAC with similar radiological features (30). Also, due to the rarity of these PM-SCLC, there are no recommended guidelines or strategies for the most appropriate management (12, 24, 29). It has been reported that patients with PM-SCLC are often in the advanced stage of disease, with poor general condition or systemic metastasis, and low postoperative survival rate, and surgical treatment is not recommended (13, 31). Isabel et al. showed that chemotherapy could be considered in the case of primary tumor synchronous PM (13, 32), which was a safe and effective treatment decision that could improve survival in selected patients. Thus, in our study, we analyzed and compared the characteristics of PM-SCLC and PDAC, and aimed to establish a preoperative a noninvasive and convenient nomogram diagnostic predictive model to provide a basis for optimal treatment decisions for the subsequent surgical treatment and adjuvant therapy.

In the present study, we developed three models based on clinical characteristics, radiological features and biomarkers for diagnostic prediction of PM-SCLC. We identified 3 clinical characteristics (clinical symptoms, CA199, gender) in the clinical model and 6 radiological features (pancreatic duct dilatation, pushed pancreatic duct, tumor size, parenchymal atrophy, vascular involvement, enhancement type) as significant in the CT imaging model. The combined model with the fusion of clinical, radiological features, and biomarkers was proven to have the optimal performance in distinguishing PM-SCLC and PDAC, with the value of sensitivity, specificity, accuracy, and AUC were 0.945, 0.832, 0.868, and 0.950, respectively. Furthermore, based on the optimal feature subsets (clinical symptoms, CA199, tumor size, parenchymal atrophy, vascular involvement and enhancement type) of the training cohort, a nomogram diagnostic predictive model for PM-SCLC was constructed. DCA, a method available to obtain net benefit based on threshold probability, revealed the superiority of the nomogram in the classification between PM-SCLC and PDAC. To validate the stability and reliability of all models, further verification was applied in the internal validation cohort and an independent external validation cohort, with the value of AUC were 0.928 and 0.976, respectively, the nearly similar values of AUC indicating the excellent robustness and generalization, meaning good practical value for diagnostic predictive model for PM-SCLC.

Previous studies (32, 33) have suggested that clinical symptoms of PM-SCLC and PDAC were similar including weight loss, jaundice, pain, dyspepsia and nausea, etc. The clinical symptoms of PM-SCLC were not prominent except in a few cases, while most PDAC is accompanied by clinical symptoms except for early PDAC (30, 32, 34, 35). In our study, the absence of clinical symptoms was discriminating variable to differentiate PPM from PDAC, there were significant differences in clinical symptoms between the two groups (P < 0.001). CA199 plays an important role in the development of PDAC and may be used as a biomarker for the diagnosis and/or prognosis of PDCA (23, 24, 36). Zhang et al. reported that CA199 performs better in symptomatic patients, with a sensitivity and specificity of 79% to 81% and 82% to 90% respectively for the diagnosis of PDCA in this setting (37, 38), but studies on the correlation between CA199 and PM have not been reported at home and abroad. In our study, patients with CA199 positive accounted for 40% (22/55) of PM-SCLC and 79% (94/119) of PDCA in training cohort. CA199 negative was another discriminating variable to differentiate PPM from PDAC, there were significant differences in CA199 between the two groups (P = 0.003).

In our study, the age (mean (SD)) of PM-SCLC in the three cohorts was 60.0 (8.6), 59.8 (9.4), 62.2 (9.3) years, respectively, which was not statistically significant compared to PDCA and was similar to previous studies (26). The final study population consisted of 93 patients with PM-SCLC, 74 men and 12 women, which was not statistically significant compared to PDCA. Because small cell lung cancer (SCLC) usually occurs in older men (60-70 years of age) and has the worst prognosis due to the rapid growth of disseminated features before the first diagnosis (39, 40). In addition, similar tendencies were observed for CEA value, CA125 value, CA199 value, tumor size, number, tumor shape, retention cyst, peripancreatic fluid, peritumoral lymph nodes, enhancement pattern, enhancement degree, enhancement type, enhancement type1 between the two groups, respectively, in the three cohorts, although not always statistically significant in univariate analysis (**Table 2**).

Multivariate logistic regression analyses were performed to obtain six radiological features for PM-SCLC diagnosis in CT imaging model or combined model (**Tables 4**, **5**). The six radiological features were all independent risk factors, including pancreatic duct dilatation, pushed pancreatic duct, tumor size, parenchymal atrophy, vascular involvement and enhancement type, which were similar to those reported in previous studies (18, 26, 41). Although pancreatic duct dilatation wasn’t obtained into the combined model, both Galia, et al. (18) and Barat, et al. (26) confirmed that absence of pancreatic duct dilatation were the discriminating features for the diagnosis of PM-SCLC against PDAC. In our analysis, pushed pancreatic duct was another independent predictor not obtained into the combined model and not reported in previous studies, but presence of pushed pancreatic duct accounted for 35.5% (33/93) of PM-SCLC and 0.4% (1/206) of PDCA in our datasets. There was statistical difference between two groups, reminding us to pay attention to it and perform further exploration in the future. PM-SCLC arises from pancreatic parenchyma and PDAC arises from ductal epithelial cells. Therefore, PM-SCLC does not easily cause the pancreatic duct to dilate, but rather pushes or compresses the pancreatic duct. In previous report (26, 42), pancreatic duct dilatation could cause upstream parenchymal atrophy and this finding was observed in 5/34 patients (5%), and significantly less frequently in the group of PM-SCLC. Thus, absence of parenchymal atrophy was the discriminating features for the diagnosis of PM-SCLC against PDAC.

We observed that the largest tumor diameter of PM-SCLC (28.1 (18.1)mm, 21.3 (13.0)mm, 27.2 (16.4)mm) was smaller than that of PDAC (33.5(13.1)mm, 38.5(16.1)mm, 33.0(13.4)mm) in the three cohorts, which was a statistically significant difference compared to PDCA and was different from previous studies. Barat, et al. (26) showed that no differences in largest tumor diameter were found between PM (35.0 ± 21.1 mm) and PDAC (32.1 ± 9.2 mm). In the Tsitoutridis et al. study, mean diameter of PM was 2.75 mm (range: 12-52 mm) (43) and 32.2 mm (range: 11-81 mm) in the Shi et al. Study (44). This was because the primary tumors of these PM were multiplicity. In this study, we found little vascular involvement in PM-SCLC, accounting for only 10.8% (10/93) of cases. Conversely, vascular invasion could be seen frequently in PDAC (23), accounting for 64.1% (132/206) of cases. As reported by Low et al. this sign was closely related to the lymphatic drainage pathway of the pancreas (45). The absence of vascular involvement was a frequent feature of PM-SCLC, in line with previous studies (26). Moreover, among more recent studies, enhancement type were the discriminating features for the diagnosis of PM-SCLC against PDAC, were similar to ours. PM-SCLC and PDAC are poorly vascularized tumors, and low or equal-attenuation on all imaging phases (unenhanced, pancreatic phase and portal venous phase) on CT (46). But the enhancement type of PM-SCLC was mainly gradual or Fast forward and backward, while PDAC was mainly strengthened by progressive, in our study.

However, our current study had several limitations. First, the data were not sufficient, especially the sample size of the independent external validation cohort was particularly small, which may have caused some bias. Thus, a larger-scale trial is required. Second, of all the PM-SCLC cases eventually included in our study, some were diagnosed by CT follow-up but not confirmed by further pathology and/or histology. This would weaken the confidence but would not affect the final prediction. Third, the patients of the established models in our study had PM-SCLC including single nodule or multiple nodule patterns, and thus the significance of differentiating isolated PM-SCLC from PDAC is unclear, and a large sample of isolated PM-SCLC is required. Fourth, in our study, the pancreatic examination protocol did not include pancreatic MR Enhancement. Finally, due to the inherent defects of imaging including resolution and subjective diagnosis, texture analysis of model building is needed in our further studies.

## Conclusions

In brief, we successfully constructed a noninvasive and convenient nomogram diagnostic predictive model based on clinical characteristics, radiological features and biomarkers to accurately preoperatively differentiate PM-SCLC from PDAC. Moreover, the nomogram diagnostic predictive model demonstrated a good predictive performance. This model may help to provide a basis for optimal treatment decisions for the subsequent surgical treatment and adjuvant therapy.

## Data availability statement

The datasets presented in this study can be found in online repositories. The names of the repository/repositories and accession number(s) can be found in the article/supplementary material.

## Ethics statement

The studies involving human participants were reviewed and approved by the ethics committee of The Second Affiliated Hospital of Zhejiang University School of Medicine (Approval numbers 20220989) and the Second Affiliated Hospital of Zhejiang Chinese Medical University (Approval numbers 2022-LW-020-01). Written informed consent for participation was not required for this study in accordance with the national legislation and the institutional requirements. Written informed consent for participation was not required for this study in accordance with the national legislation and the institutional requirements.

## Author contributions

J-XX conceived the study and drafted the manuscript. R-SY contributed to the supervision of the whole process. J-BH and X-YY helped critically revise the manuscript for important intellectual content. NF and X-SH carried out to collect the data of patients. X-ZZ and Q-PR helped in images analysis. Y-GW were responsible for writing code and data analysis. All authors contributed to the article and approved the submitted version.
